# Clinical Presentation of Atopic Dermatitis by Filaggrin Gene Mutation Status during the First 7 Years of Life in a Prospective Cohort Study

**DOI:** 10.1371/journal.pone.0048678

**Published:** 2012-11-15

**Authors:** Charlotte Giwercman Carson, Morten Arendt Rasmussen, Jacob P. Thyssen, Torkil Menné, Hans Bisgaard

**Affiliations:** 1 Copenhagen Prospective Studies on Asthma in Childhood; COPSAC, Health Sciences, University of Copenhagen, Copenhagen University Hospital, Gentofte, Copenhagen, Denmark; 2 Faculty of Life Sciences, University of Copenhagen, Frederiksberg, Denmark; 3 National Allergy Research Centre, Department of Dermato-Allergology, Copenhagen University Hospital Gentofte, Hellerup, Denmark; CNRS-University of Toulouse, France

## Abstract

**Background:**

Filaggrin null mutations result in impaired skin barrier functions, increase the risk of early onset atopic dermatitis and lead to a more severe and chronic disease. We aimed to characterize the clinical presentation and course of atopic dermatitis associated with filaggrin mutations within the first 7 years of life.

**Method:**

The COPSAC cohort is a prospective, clinical birth cohort study of 411 children born to mothers with a history of asthma followed during their first 7 years of life with scheduled visits every 6 months, as well as visits for acute exacerbations of dermatitis. Atopic dermatitis was defined in accordance with international guidelines and described at every visit using 35 predefined localizations and 10 different characteristics.

**Results:**

A total of 170 (43%) of 397 Caucasian children developed atopic dermatitis. The R501X and/or 2282del4 filaggrin null mutations were present in 26 (15%) of children with atopic dermatitis and were primarily associated with predilection to exposed skin areas (especially the cheeks and back of the hands) and an up-regulation of both acute and chronic dermatitis. Furthermore, we found the filaggrin mutations to be associated with a higher number of unscheduled visits (3.6 vs. 2.7; p = 0.04) and more severe (moderate-severe SCORAD 44% vs. 31%; p = 0.14), and widespread dermatitis (10% vs. 6% of the body area, p<0.001) with an earlier age at onset (246 vs. 473 days, p<0.0001) compared to wild-type.

**Conclusion:**

In children, filaggrin mutations seem to define a specific endotype of atopic dermatitis primarily characterized by predilection to exposed areas of the body, in particular hands and cheeks, and an up-regulation in both acute and chronic morphological markers. Secondary, this endotype is characterized by an early onset of dermatitis and a more severe course, with more generalized dermatitis resulting in more frequent medical consultations.

## Introduction

Approximately 10% of the general population carry at least one null mutation in the filaggrin gene (*FLG*) [1]. Normal gene expression results in intracellular filaggrin proteins which aggregate keratin filaments, leading to keratinocyte compaction and formation of the stratum corneum. Also, filaggrin expression is crucial for loading of lamellar body contents, uniform extracellular distribution of secreted organelle contents, and correct lamellar bilayer architecture; key features in upholding the function of the two compartment cornified envelope that prevents transepidermal water loss and penetration of micro-organisms, chemicals and allergens [2], [3]. Heterozygous, and in particular homozygous or compound heterozygous carriers of *FLG* null variants, experience dry, scaly and fissured skin more often that non-mutation carriers [4], [5] and recent *in vivo* measurements of the stratum corneum in patients with AD also found lower levels of natural moisturizing factor among *FLG* deficient patients [6], [7]. Furthermore, *FLG* mutations are major predisposing factors for atopic dermatitis (AD) [1], [8]–[11] and are associated with early onset of AD, persistence of AD into adulthood and asthma and allergic sensitization [8], [9], [12]–[14]. Mutations seem to predict dose-dependent alterations in epidermal permeability barrier function and in accordance with this, homozygous carriers typically develop ichtyoisis vulgaris and/or AD very early in life, whereas heterozygous carriers may experience a milder course or no symptoms at all [3], [15], [16].

Phenotyping based on *FLG* null mutations might represent a novel endotype with known underlying molecular causes and, presumably, distinct clinical features. Endotyping of AD is important for segmentation of patients and future investigations of individualized treatment possibilities. Phenotypical characterization based on prospective data collection has not yet been reported for the *FLG* mutations. We meticulously characterized the pattern of AD in the Copenhagen Prospective Birth Cohort during the first 7 years of life and performed stratification by *FLG* mutation status.

## Materials and Methods

### Ethics Statement

The Copenhagen Study on Asthma in Childhood (COPSAC) was conducted in accordance with the guiding principles of the Declaration of Helsinki, approved by the Ethics Committee for Copenhagen (KF 01-289/96) and The Danish Data Protection Agency (2002-41-2434). Data validity was assured by compliance with “Good Clinical Practice” (GCP) guidelines and quality control procedures. Data were collected on-line and locked after external monitoring and an audit trail was run routinely. Informed written consent was obtained from all parents.

### Participants

The Copenhagen Study on Asthma in Childhood (COPSAC) is a prospective, clinical, birth cohort study including 411 children born to mothers with asthma. The children were enrolled at one month of age and visited the clinical research unit at scheduled visits every six months as well as for any acute skin symptoms. The main recruiting area of the cohort was greater Copenhagen, Denmark and all children were born between August 1998 and December 2001. The study was previously detailed [17]–[19]. In this study, we included data from Caucasians only, i.e. 397 of 411 enrolled children. Among these, 172 (43%) were diagnosed with AD before age 7 years. In two cases, information about *FLG* mutation status and registration of dermatitis were missing, leaving 170 children for analysis. Two homozygous/compound heterozygous children were grouped with the heterozygous children. Skin examinations, diagnoses and treatment of dermatitis were handled by medical doctors employed for this purpose in the clinical research unit.

### Risk Assessments

AD was stratified by *FLG* mutation status and the groups were analyzed for differences during the first 7 years of life. AD was defined based on the criteria of Hanifin and Rajka [20]. *FLG* genotyping was performed for R501X and 2282del4 mutations using TaqMan-based allelic discrimination assay and fluorescently labelled PCR (Applied Biosystems 3100, 3730 and 7700 sequence detection system, Foster City, CA, USA) [8]. Individuals designated as “wild type” were therefore patients not carrying these two common mutations, but carrier status of other, rare *FLG* mutations, cannot be excluded for this group.

At each visit, the following observations were registered:

- **Anatomical localization** of dermatitis lesions were divided into 35 predefined areas: abdomen, ankle (back), ankle (front), back (lower), back (upper), cheek, chest, chin, ear, elbow (back), elbow (front), eye area, foot (back), foot (sole), forearm (back), forearm (front), forehead, hand (back), hand (palm), knee (back), knee (front), lower leg (back), lower leg (front), nappy region, neck (back), neck (front), nose, perioral, scalp, upper arm (back), upper arm (front), upper leg (back), upper leg (front), wrist (back) and wrist (front).
- **Morphology** of the single dermatitis lesion was based on the following characteristics: area (in percent of the total body surface area), erythema, lichenification, crusts, dryness, vesicles, squamation, fissures, edema and excoriations (graduated from 0 (none) to 3 (severe)).
- **Severity** of the dermatitis episode was assessed using the Scoring Atopic Dermatitis index (SCORAD) [21].

### Statistical Analyses

Associations between *FLG* mutation status (null vs. wild type) and different variables were investigated using the following analyses: Cox proportional-hazards regression (PROC PHREG) (AD diagnosis), chi-square test (PROC FREQ) (frequency of localizations), log-rank test (PROC LIFETEST) (age at onset of AD), the non-parametric Wilcoxon Rank-Sum Test (PROC NPAR1WAY) (number of visits in the clinical research unit), and GEE-model (PROC GENMOD) (SCORAD, total area of the body involved pr visit). All analyses were done in SAS version 9.1 (SAS Institute Inc, Cary, NC). The overall significance level used was 0.05.

A multivariate approach was employed to detect clinical patterns related to *FLG* null mutation status. We applied Partial Least Squares Discriminant Analysis (PLSDA) [22] to describe the anatomical location of dermatitis and Principal Component Analysis (PCA) to describe dermatitis morphology, in both cases with the aim of exploring differences in relation to *FLG* mutation status.

#### 1) Anatomical location

The number of registrations (continuous) was used as predictors. The pattern of the 35 different regions was visualized including how they were associated with the *FLG* genotype both individually and compared to the other regions. The component consists of a *score matrix* with samples distribution and a *loading matrix* where the relation between the predictors (regions) was created and displayed.

#### 2) Dermatitis morphology

For each region (n = 35) and each *FLG* mutation type (wild type and null), the morphology parameters (erythema, lichenification, crusts, dryness, vesicles, squamation, fissures, edema and excoriations) were represented by a weighted average across all registrations taking the different registration frequencies into account. This resulted in a 70 by 9 matrix. PCA with two components and varimax rotation was then conducted on this matrix. Here the *score plot* displays the similarity between regions in connection with *FLG* mutation status. The *loading plot* unravels the correlation structure between the morphology parameters [23].

PLSDA and PCA were conducted using the PLStoolbox ver. 6.0.1 (Eigenvector Inc, Manson, Washington, USA.). In addition in-house algorithms were used for visualization. All analysis where conducted in Matlab® R2010b version 7.11.0.584.

Additional methodological details are given in “Supporting Information S1: Materials and methods”.

## Results

Twenty six (15.3%) of 170 AD children and 15 (7.1%) of 212 non-AD children were *FLG* null mutation carriers (HR 2.23, 95% CI 1.47–3.39, p = 0.0002). The lifetime prevalence of AD was 63% in children with the *FLG* null genotype and 43% in children with the wild type.

### Anatomical Dermatitis Localizations

**Figure 1:**
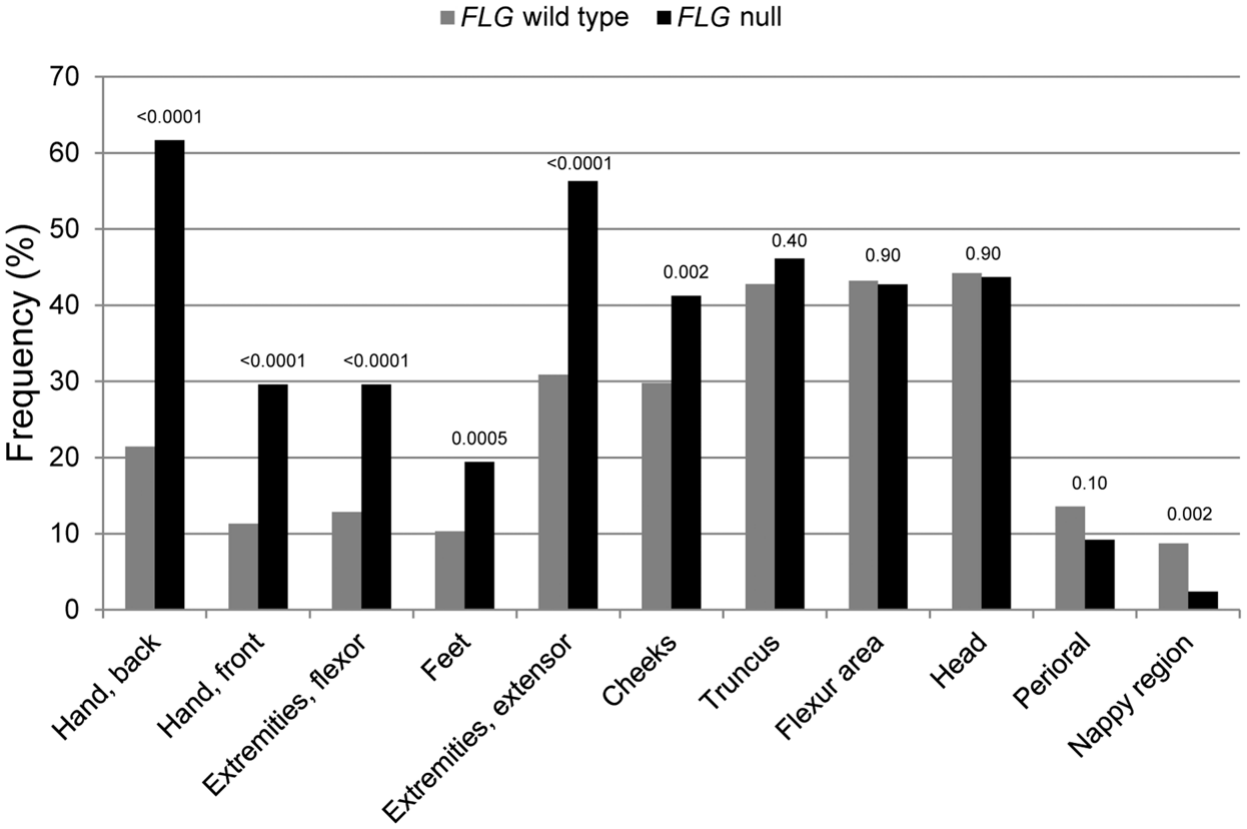
Frequency of skin localizations in respect to filaggrin status. Frequency of localizations in relation to the number of visits at the clinical research unit, 0–7 years, grouped and stratified by *FLG* status. Numbers above the bars indicate the p values.

**Figure 2:**
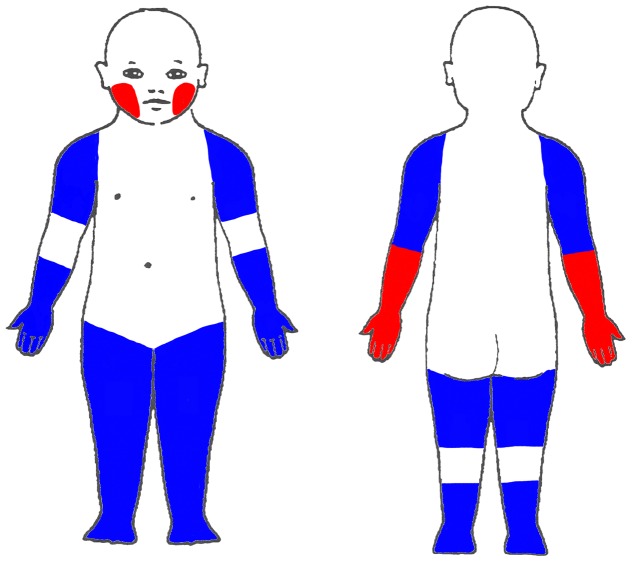
Summary of skin localizations in respect to filaggrin status. The figure summarizes the localizations more often affected in children with *FLG* null mutations compared to wild type children (red and blue areas), with red areas illustrating the localizations specifically selected by PLSDA as driving sites.

The anatomical localization of dermatitis stratified by *FLG* mutation status (affected per visit) is presented in figure 1. Here, the 35 predefined areas were grouped into 11 areas (for details, please see Table S1). The analysis showed that involvement of the palm and back of the hands, the flexor and extensor extremities, the feet and the cheeks was statistically significant associated with the *FLG* null genotype (p values between <0.0001 and 0.002). PLSDA analysis of the original 35 regions confirmed this positive association, with a tendency towards higher number of different localizations for the *FLG* null children, as all loading values were positive in the first component, mainly driven by affection of the cheeks and the back of the hands, No differences were seen for the flexural area (p<0.01) (Figure S1). Figure 2 summarizes the localizations that were more often affected in *FLG* null children (red and blue areas), with the red areas illustrating the localizations significantly selected by the PLSDA (cheeks and back of the hand). To investigate whether the anatomical localizations associated with the *FLG* null genotype were age dependent, further stratification was performed (0–3 and 3–7 years). However, we found no change in predilection of dermatitis (data not shown).

### Morphology

**Figure 3:**
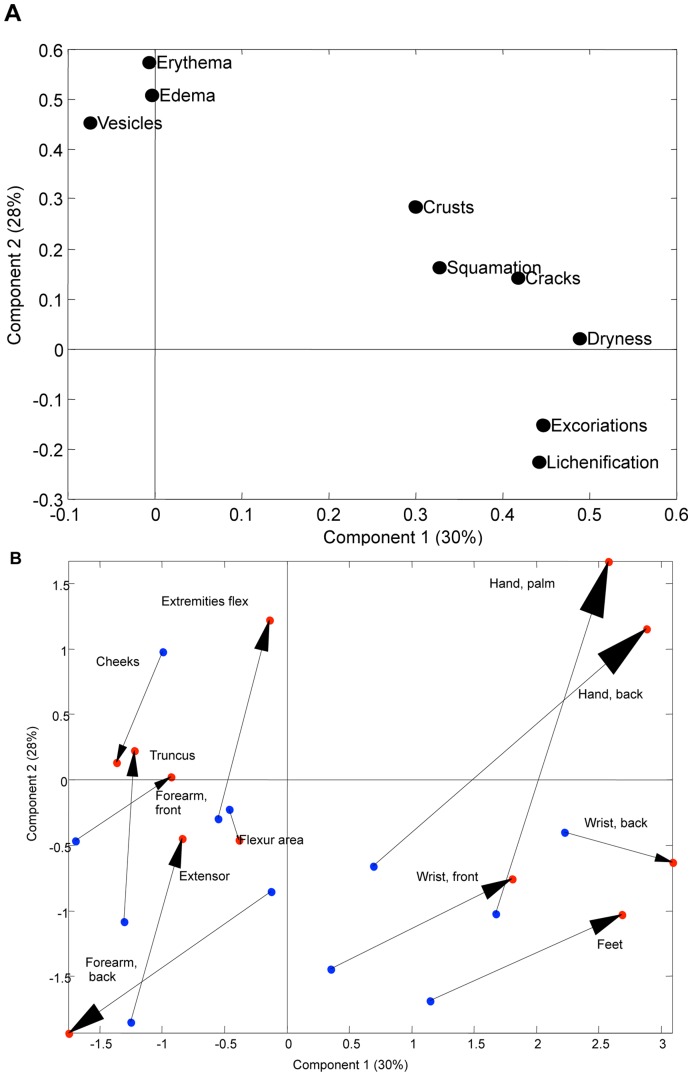
Morphology of the individual skin localizations. A: PCA loading plot for the first two components. Morphological characteristics such as fissures, lichenification and crust were found correlated as seen by the grouping at the x-axis (component 1, chronic markers), whereas edema, erythema and vesicles were grouped at the y-axis (component 2, acute markers). **B:** PCA score plot showing the morphology of the individual localizations. Each region is shown as two points (“blue circles”: *FLG* wild type, “red circles”: *FLG* null) connected with an arrow from *FLG* wild type to *FLG* null. E.g. hand (back) obtained higher values with respect to chronically inflammatory markers than e.g. forearm (front). A general up-regulation of both acute and chronic markers was observed for *FLG* null children across almost all regions.

Firstly, three regions were excluded from the analyses due to a low number of registrations (eye area, plantar part of the foot, and the nose). Secondly, two components, describing 58% of the total variation, were extracted by PCA (component 1 and 2). The PCA loading plot separated chronic signs of dermatitis such as fissures, lichenification and crusting (x-axis) from acute signs of dermatitis such as oedema and erythema (y-axis). Characteristics closely positioned are correlated and therefore exhibited similar pattern, e.g. high loadings for erythema track with high loadings for edema and vesicles etc. (Figure 3a). It is important to notice that the PCA does not aim at separating different markers (unsupervised), and that the chronic and acute components simply appear, because the main variation turns out to be reflected by these two patterns. Thirdly, the information obtained in the PCA loading plot, was combined with information about the morphology at the separate skin localizations via a score plot (figure 3b). At the score plot, the positions in truncated principal component space of the dermatitis lesions are shown. The figure describes the distribution of the skin regions, so that closely positioned points exhibit a similar pattern with respect to the morphology parameters reflected in the loading plot (figure 3a). Each region is shown as two points (blue and red circles representing *FLG* wild type and *FLG* null, respectively) connected with an arrow from the *FLG* wild genotype to the *FLG* null genotype. To get an idea of how these two plots are interpreted, consider the skin region *Extremities flex* as an example. The location of *FLG* null is high in component 2 compared to *FLG* wild type (figure 3b). This means that this skin region exhibits a more severe dermatitis for *FLG* null with respect to the acute inflammatory markers, that span this component (component 2– figure 3a). On the other hand, there seems to be no difference between the severities of the chronic inflammatory markers between the two genotypes reflected by the position on component 1. Likewise, it is registered that *Wrist back,* in terms of chronic inflammatory markers, is one of the skin regions with the most severe dermatitis with slightly higher severity for the *FLG* null type compared to the *FLG* wild type, but with no difference between the severities of acute markers (component 2). Figure 3b showed that the majority of arrows pointed up or towards the right, i.e. pointing in the same directions as the acute and chronic markers at the loading plot, which means that a general up-regulation of both acute and chronic markers was observed in *FLG* null children when compared to wild type children. Interpretation of single region differences between the two groups, revealed a pattern with certain regions up-regulated in acute markers (extensor areas, extremities flex areas, truncus, hand (palm/back)) and in chronic markers (feet area, wrist (front/back), hand back). These two figures (3a and 3b), serve as a comprehensive overview comparing the severity pattern of nine dermatitis markers for 15 different skin regions between two *FLG* genotypes. At figure 3b the localizations are grouped according to Table S1 except for the two groups *Hand area front* and -*back* which are kept as (six) individual regions. For the plot based on the original 32 skin regions, see Figure S2.

### Severity

The skin was examined at 905 separate visits of which 276 (30.5%) were unscheduled visits for acute skin symptoms. Children with the *FLG* null mutations had more unscheduled visits than wild type children (mean 3.6 vs. 2.7 visits, p = 0.036) and *FLG* null children presented a higher SCORAD score than wild type children, albeit not statistically significant (44% vs. 31% with moderate-severe SCORAD, p = 0.14). Similar, non-significant trends were found in SCORAD values at unscheduled and scheduled visits separately (results not shown).

*FLG* null carriers had earlier onset of dermatitis (median age 128 vs. age 299 days, p<0.0001) and a more widespread dermatitis at each visit compared to wild type children (median 10% vs. 6% of the body, p<0.001). A similar difference was seen at both scheduled and unscheduled visits (results not shown).

## Discussion

### Main Finding

Primarily, this study showed that in our cohort of at-risk children, the *FLG* null driven AD endotype is characterized by dermatitis at anatomical localizations that tend to be exposed to drying conditions (e.g. wind, cold, sun and radiations from indoor heating), especially the back of the hands and the cheeks (figure 2) and with dermatitis lesions characterized by an up-regulation in both acute and chronic markers when compared to wild type children. Moreover, this endotype had a higher frequency of acute dermatitis episodes, as illustrated by more unscheduled visits to our clinic, and also a more generalized, and severe dermatitis (higher SCORAD) when compared to *FLG* wild type children with AD, although the differences in severity were marginal.

### Strengths and Limitations

The COPSAC cohort consists of prospective collection of data during the first 7 years of life. The diagnosis, detailed phenotype and management of skin lesions have been controlled solely by the clinical research unit physicians from standard operating procedures and treatment algorithms, and not by the general practitioner or others. Hence, the specificity of the AD diagnosis is high and the risk of misclassification is expected to be low. This is of particular importance in the clinical evaluation of AD where inter-observer variation may be a problem [24]. The children were followed every 6 months and were also seen in case of acute flare-up of dermatitis. Such prospective data collection reduces the risk of recall bias.

Our study is limited by all children being of Caucasian descent and it is possible that *FLG* mutations may not exert the same effect in other races. Furthermore, they are at-risk children with mothers suffering from asthma, thereby risking interaction with asthma genetics. Therefore, our results need replication in an unselected population.

Because of the project protocol, with the children being followed closely by trained physicians when having acute flare-ups, these children are more likely to receive better treatment than the general population. This optimized care is a potential confounder, which may dilute the differences between *FLG* null and wild type children with AD. However, this would support the genuine difference between the two groups regarding the anatomical localization and morphological presentation of their dermatitis. Despite our modest sample size we are the first to report a prospective registration of the dermatitis morphology and localization in AD patients stratified by *FLG* mutations. Since we find differences between the two strata, study size did not cause a type 2 error.

We used classic bar charts to illustrate our main findings and confirmed them by multivariate pattern analysis, i.e. PLSDA and PCA. This approach facilitates the handling of many variables in the same analysis, which is especially important for complex phenotypic data. Multivariate pattern analysis makes it possible to study the systematic variation, while filtering out uncorrelated random variation and hence observe patterns not otherwise recognized by traditional univariate statistical analysis. We were able to extract plausible acute and chronic components from the morphological data and characterize different skin regions. In this way, we use a data analytical approach more in line with the clinicians approach to clinical problems which is more often characterized by pattern recognition rather than any single markers.

### Interpretation

AD has traditionally been classified as acute vs. chronic, intrinsic vs. extrinsic, associated with ichthyosis vulgaris, based on morphology (nummular, atopic prurigo, lichen planus-like, pityriasis alba) or based on localization (hand, juvenile plantar and palmar, eyelid, cheilitis, nipple, periorificial). However, none of these classifications seem to be satisfying, because patients often experience dynamic changes between the suggested categories and with affection of different sites [25]. Our data suggest that *FLG* null mutations, the cause of ichthyosis vulgaris, are associated with dermatitis at “exposed areas”, especially the cheeks and the back of the hands, but also the feet and extensor areas. These sites are typically exposed to drying and irritant factors such as water, sun, wind, child’s play, changes in temperatures and radiation from indoor heating. It is likely that these factors may act as local triggers, resulting in further dry skin than dictated by the inherited skin constitution, and then dermatitis. In contrast, dermatitis in wild type children was not located on these rather exposed sites. Supporting this interpretation, *FLG* deficient flaky tail mice bred in cages with contact to the environment, had a higher level of dermatitis and skin inflammation when compared to mice bred in cages with no environmental contact [26]. Other mechanisms, rather than primary barrier abnormality, might be responsible for the *FLG* wild type children’s dermatitis. This could be changes in immunomodulatory factors, heat or bacterial growth.

Our study’s suggested association between *FLG* null carrier status and hand dermatitis, is in line with a recent general population study, showing that the *FLG* null genotype significantly increased the risk of hand dermatitis in individuals with AD and that it was associated with early onset (before age 6) and persistence into adulthood [27]. Furthermore, the clinical observation was recently made, that adult patients with the *FLG* null genotype had a distinct phenotype of hand dermatitis characterized by fissured eruptions on the back of the hands and wrists with only sparse involvement of the palmar aspects, similar to the clinical description of hand dermatitis among atopic individuals previously described by other groups [28], [29]. It is interesting that we could partly confirm this finding in our cohort of children, as *FLG* null carriers mainly had chronic markers of dermatitis on the back of their hands, whereas acute markers were upregulated in the palms as well as the back of the hands. Hence, the “dorsal hand eczema” as seen in patients with AD might indeed be associated with the *FLG* null genotype.

As a secondary finding we showed that *FLG* null children had more severe dermatitis, characterized by a higher number of visits in the clinical research unit, a more generalized dermatitis and a non-significant trend of higher SCORAD value. Also, *FLG* null mutations were associated with an earlier onset of disease. These observations are in agreement with previous cross-sectional reports [12], [13], [25], [30] and suggest that *FLG* genotyping should be considered in the initial diagnostic work of patients suspected with AD and that it may be useful for classification.

### Conclusion

In this study of at-risk children with AD, we find that the *FLG* null defines an endotype characterized by dermatitis with a predilection site at exposed areas of the body, in particular hands and cheeks, and an up-regulation in both acute and chronic morphological markers. Furthermore this endotype is characterized by an early onset of dermatitis and a more severe course with more generalized dermatitis resulting in more frequent medical consultations. These findings will hopefully help us segmenting AD patients and promise individualized treatment in the future, as well as improved disease prediction and research into novel preventive approaches. This is the first study reporting a prospectively characterizing of the morphology and localization of childhood dermatitis and stratifying it by *FLG* mutation status. However, future studies including replication in an unselected population are needed to confirm our findings.

## Supporting Information

Figure S1 **Figure S1A (PLSDA score plot) and Figure S1B (PLSDA loading plot): Skin localizations.** PLSDA score- (**A**) and loading (**B**) plot for having dermatitis on a given localization in relation to number of visits in the clinic. Ellipsoids are centered at population mean with half axis corresponding to the standard deviation and under normality assumption hence cover ∼50% of data. Red and blue represents *FLG* wild type and null mutation carriers, respectively.
(TIF)

Figure S2 **Morphology of the individual skin localizations.** Original PCA score plot showing the morphology of the individual localizations. Each region is shown as two points (“blue circles”: *FLG* wild type, “red circles”: *FLG* null) connected with an arrow from *FLG* wild type to *FLG* null. E.g. hand (back) obtained higher values with respect to chronically inflammatory markers than e.g. forearm (front). A general up-regulation of both acute and chronic markers was observed for *FLG* null children.
(TIF)

Table S1 Grouping the 35 predefined localizations into 11 groups.
(DOC)

Supporting Information S1 “Materials and methods” Elaborated statistical explanation for the multivariate analysis PLSDA.
(DOC)
