# Supplementary figures and images for: Clinical Presentation of Atopic Dermatitis by Filaggrin Gene Mutation Status during the First 7 Years of Life in a Prospective Cohort Study
Source: PLoS One. 2012 Nov 15;7(11):e48678. doi: 10.1371/journal.pone.0048678 (PMC3499508; doi:10.1371/journal.pone.0048678)

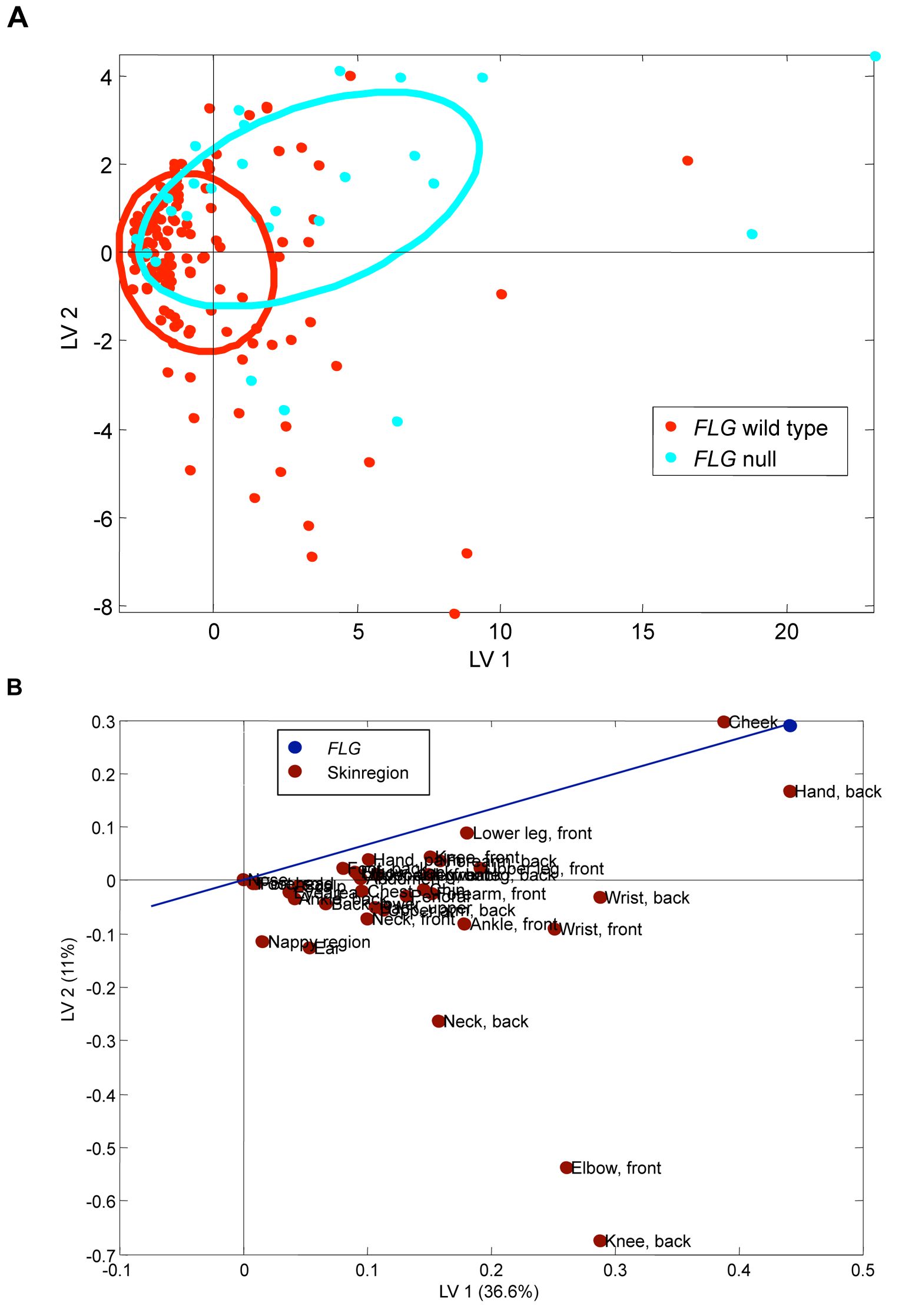

Supplement: Figure S1 — A (PLSDA score plot) and Figure S1B (PLSDA loading plot): Skin localizations. PLSDA score- (A) and loading (B) plot for having dermatitis on a given localization in relation to number of visits in the clinic. Ellipsoids are centered at population mean with half axis corresponding to the standard deviation and under normality assumption hence cover ∼50% of data. Red and blue represents FLG wild type and null mutation carriers, respectively. (TIF) [file pone.0048678.s001.tif]

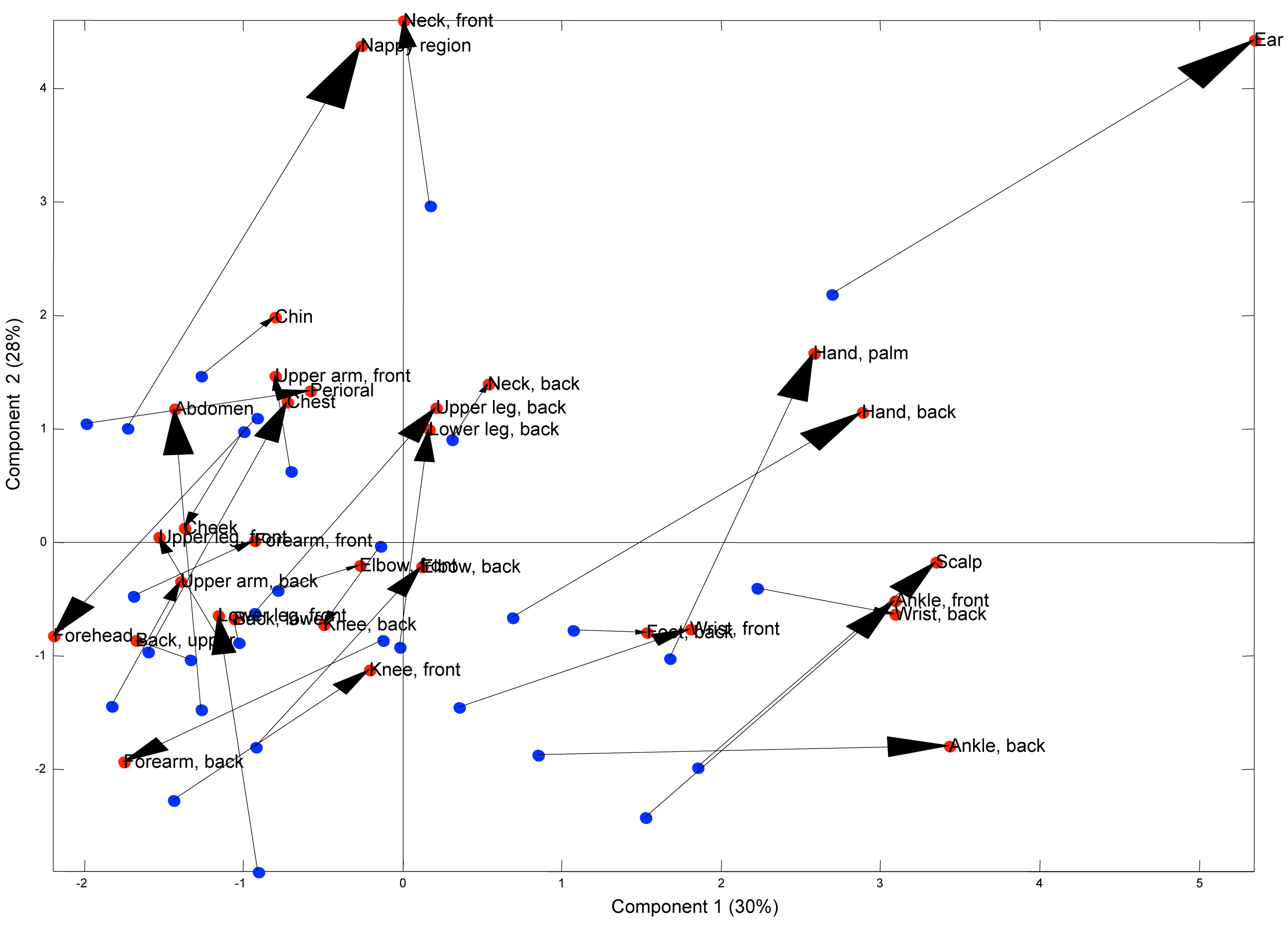

Supplement: Figure S2 — Morphology of the individual skin localizations. Original PCA score plot showing the morphology of the individual localizations. Each region is shown as two points (“blue circles”: FLG wild type, “red circles”: FLG null) connected with an arrow from FLG wild type to FLG null. E.g. hand (back) obtained higher values with respect to chronically inflammatory markers than e.g. forearm (front). A general up-regulation of both acute and chronic markers was observed for FLG null children. (TIF) [file pone.0048678.s002.tif]
