# Supplementary material for: Clinical Presentation of Atopic Dermatitis by Filaggrin Gene Mutation Status during the First 7 Years of Life in a Prospective Cohort Study
Source: PLoS One. 2012 Nov 15;7(11):e48678. doi: 10.1371/journal.pone.0048678 (PMC3499508; doi:10.1371/journal.pone.0048678)
Supplement: Table S1 — Grouping the 35 predefined localizations into 11 groups. (DOC) [file pone.0048678.s003.doc]

**Supporting information table S1:** Grouping the 35 predefined localizations into 11 groups.

| **Truncus** | **Flexur area** | **Hand area, back** | **Hand area, front** | **Head** | **Cheeks** | **Extremities, extensor** | **Nappy region** | **Perioral** | **Feet** | **Extremities flexor** |
| --- | --- | --- | --- | --- | --- | --- | --- | --- | --- | --- |
|
| Abdomen | Elbow, front | Hand, back | Hand, palm | Chin | Cheek | Elbow, back | Nappy region | Perioral | Foot, back | Lower leg, back |
| Back, lower | Knee, back | Wrist, back | Wrist, front | Ear |  | Knee, front |  |  | Foot, sole | Upper arm, front |
| Back, upper |  | Forearm, back | Forearm, front | Eye area |  | Lower leg, front |  |  | Ankle, back | Upper leg, back |
| Chest |  |  |  | Forehead |  | Upper arm, back |  |  | Ankle, front |  |
|  |  |  |  | Nose |  | Upper leg, front |  |  |  |  |
|  |  |  |  | Scalp |  |  |  |  |  |  |
|  |  |  |  | Neck, back |  |  |  |  |  |  |
