# Supplementary material for: Clinical Presentation of Atopic Dermatitis by Filaggrin Gene Mutation Status during the First 7 Years of Life in a Prospective Cohort Study
Source: PLoS One. 2012 Nov 15;7(11):e48678. doi: 10.1371/journal.pone.0048678 (PMC3499508; doi:10.1371/journal.pone.0048678)
Supplement: Supporting Information S1 “Materials and methods” — Elaborated statistical explanation for the multivariate analysis PLSDA. (DOC) [file pone.0048678.s004.doc]

**Supporting information S1:** Materials and methods

*Statistical analyses*

We applied Partial Least Squares Discriminat Analysis (PLSDA) and principal component analysis (PCA) which may be regarded as extensions of the multiple linear regression model (y=ax+b). In the latter model, one can make reasonable estimates (predictions) for new observations based on the linear relationship between variables (e.g. between height and weight). The PLS is to some degree related to the linear regression model as it also aims to identify a linear model based on observed and expected values, but in contrast, these variables are projected into a new space. The PLS model can be regarded as a dimension reduction approach that is coupled with a regression model. It is typically used in situations where the matrix of predictors has more variables than observations or when there is multicollinearity among the x-values (this is when two or more predictor variables are highly correlated). The PLS model has for these reasons also been used for analysis of high-dimensional genomic data where expression of thousands of genes is evaluated. In general, PLS can be regarded as a robust statistical model suited for a large number of variables, which also takes into account the interdependencies that may exist among the variables.
